# Coalition Formation Based Compressive Sensing in Wireless Sensor Networks

**DOI:** 10.3390/s18072331

**Published:** 2018-07-18

**Authors:** Alireza Masoum, Nirvana Meratnia, Paul J. M. Havinga

**Affiliations:** Pervasive Systems, Department of Computer Science, University of Twente, Drienerlolaan 5, 7522 NB Enschede, The Netherlands; n.meratnia@utwente.nl (N.M.); P.J.M.Havinga@utwente.nl (P.J.M.H.)

**Keywords:** compressive sensing, coalition, sparsity, belief propagation, joint sparse recovery

## Abstract

Compressive sensing originates in the field of signal processing and has recently become a topic of energy-efficient data gathering in wireless sensor networks. In this paper, we propose an energy efficient distributed compressive sensing solution for sensor networks. The proposed solution utilizes sparsity distribution of signals to group sensor nodes into several coalitions and then implements localized compressive sensing inside coalitions. This solution improves data-gathering performance in terms of both data accuracy and energy consumption. The approach curbs both data-transmission costs and number of measurements. Coalition-based data gathering cuts transmission costs, and the number of measurements is reduced by scheduling sensor nodes and adjusting their sampling frequency. Our simulation showed that our approach enhances network performance by minimizing energy cost and improving data accuracy.

## 1. Introduction

Energy efficiency is a continuing concern within wireless sensor networks. Every sensor network is operational as long as they have enough energy resources. Therefore, to ensure the longevity of a network, energy efficient techniques are essential [1]. Compressive sensing is one of the new energy efficient compression based data gathering techniques that is introduced in recent years.

Compressive sensing is a concept originated from the field of signal processing. The promise of compressive sensing is that it can reconstruct sparse or compressible signals from a small number of measurements without having a priori knowledge about the signal structure. This technique utilizes information rate instead of sampling rate to sample and recover the signal [2].

Unlike classic compression techniques, compressive sensing simplifies encoding procedures while making the decoding procedure more complicated. This characteristic is well suited for wireless sensor network data gathering applications. It employs simple encoding on the resource-restricted sensor nodes and a complex decoding procedure at the powerful base station.

Recent compressive sensing solutions proposed in wireless sensor networks have proven advantages in minimizing the number of measurements, but they are still not competitive with the existing data compression techniques [3]. Since transmission cost is the dominant energy consumption parameter, compressive sensing methods should consider minimizing the consumption by each measurement in terms of transmission cost. In some proposed solutions, each sensor node produces its measurements, aggregates these with the measurements of other nodes and transfers them to the base station. Since sensor nodes may be far from each other, the transmission cost is high enough to negate the advantages of compressive sensing-based data gathering [4]. Utilizing distributed compressive sensing could represent an important step towards improving existing compressive sensing methods in terms of energy consumption, bandwidth use and data quality [5].

In this paper, we propose a new distributed compressive sensing based data gathering approach which utilizes spatial-temporal correlation to improve the network performance in terms of energy consumption and data reconstruction accuracy. In the first step, we propose a spatial correlation based coalition formation algorithm to localize data collection of the sensor nodes. This approach leverages the sparsity distribution of signals in order to group spatially correlated sensor nodes into coalitions. Inside each coalition, we use the sparsity distribution of signals to define a utility function. This function later is used to minimize the number of active sensor nodes, which reduces energy consumption.

After coalition formation, a spatial temporal correlation based compressive sensing solution is proposed inside each coalition. The proposed solution employs a block diagonal measurement matrix to produce a linear combination of sensor node readings. The block diagonal measurement matrix is structured in a way that balances the computation and communication load over the coalitions. This spatial-temporal correlation based compressive sensing is used inside each coalition to compress sensor node readings and transfer it to the base station. Upon receiving compressed data, the base station applies a joint sparse signal recovery mechanism to reconstruct compressed data. In the first step, a joint sparse signal recovery is run to find the common sparsity profile among the coalitions. This recovery procedure then executes inside each coalition to achieve a common profile among sensor nodes. Utilizing this recovery algorithm helps to achieve better accuracy in data reconstruction while the number of required measurements is reduced.

On the other hand, this paper analyses the impact of different transform basis on compressive sensing. The compressible or sparse signal may be represented in different transform bases. Choosing the appropriate transform basis is essential in sparse representation, which can lead to less measurements and more accurate signal reconstruction during the recovery phase. To the best of our knowledge, the selection of an appropriate transform basis has not been adequately studied, as most existing compressive sensing techniques have focussed on optimizing reconstruction algorithms. This paper classifies different signals based on their characteristics and investigates the assignment of an appropriate transform basis for each specific type of signal.

The remainder of this paper is organized as follows: Section 2 presents the related works while Section 3 represents network architecture. Section 4 introduces an overall overview of the proposed solution. Section 5 and Section 6 explain collation formation algorithm and data gathering procedure inside coalitions, respectively. The data reconstruction procedure is mentioned in Section 7 while Section 8 analyzes the performance of the proposed scheme. Finally, conclusions are drawn in Section 9.

## 2. Related Works

The study presented in [6] introduces a belief-propagation-based compressive sensing technique that considers the temporal correlation model of the signal as given information and employs it to reconstruct the signal. To model temporal correlation, one can decrease the number of required measurements and help to achieve better reconstruction accuracy with a specific spike-and-slab Markov model. The accuracy of this Markov model changes over time, and this can influence reconstruction accuracy. Therefore, this algorithm periodically analyzes the model parameters and employs an online estimator to predict its parameter. This estimator is based on sequential expectation maximization (EM) algorithm that is derived by maximizing the Kullback–Leibler (KL) information measure. At the end of each reconstruction period, this estimator refers to reconstructed signal coefficients for the last time period to update the Markov model parameters. This approach focusses on robust accurate data reconstruction and decreases the number of measurements. However, the energy efficiency of this solution is not addressed explicitly.

The authors of [7] discuss how to make the encoding process more efficient in terms of resource management strategy. They proposed a compressive sensing approach that considers temporal correlation among sensor node readings to recover the original signal. They combined their compressive sensing approach with gossip-based routing to recover the sparse signal in multi-hop wireless sensor networks.

In the case of telemonitoring applications, most signals, like Electrocardiography (ECG) signals, are not sparse in the time domain. Therefore, current compressive sensing solutions cannot provide accurate reconstruction. To address this problem, one study [8] addresses a block-sparse Bayesian learning-based compression technique. The proposed solution represents an extreme performance improvement in recovering non-sparse signals. Furthermore, using the block structure to recover signals lessens reconstruction complexity, which results in lower computational cost and better accuracy.

A combination of model-based data prediction and compressive sensing is addressed in [9] to achieve energy-efficient data gathering in sensor networks. Compressive sensing facilitates the compression of sensor node readings while an adaptive data prediction model is built on this basis, reducing the data sampling frequency. Using this data prediction model, sensor nodes do not require any prior information regarding monitored signals and can adapt their sampling rates to the time-varying sparsity level of the signals. Minimizing the data-sampling rate based on the sparsity level of the signal provided by compressive sensing attenuates the data-transmission rate, which conserves power.

Luo et al. [10] present a compressive data-gathering (CDG) method, which combines data compression and routing for data collection in large-scale wireless sensor networks. The result of this combination is a balanced distribution of energy drain over the network, which improves network lifetime. Furthermore, it shows a significant reduction in communication costs. With regard to scalability, this solution cannot be applied in small-scale networks and large-scale dynamic networks.

A further study [11] introduces a joint sparse signal recovery method which assumes sensor nodes have been distributed into different clusters, and uses a joint sparse signal recovery mechanism (instead of an independent reconstruction process) to recover the compressed data. This solution minimizes energy expenses in terms of data compression and transmission rates. However, it does not ensure that application-defined data accuracy requirements are met.

The authors of [12] target the event-detection application of wireless sensor networks in which the number of sensor nodes detecting an event is considerably lower than the number of all sensor nodes in the network. This may represent sparsity of events. The proposed compressive sensing-based event detection approach recovers the signal through a Bayesian reconstruction algorithm. Significant reductions in the number of measurements, as well as increased event detection probability, are the main performance achievements of this solution.

Realization and detecting an event region is an issue, which is addressed in [13]. The authors suggest a decomposition based compressive sensing (DBCS) data gathering solution for continuous monitoring of event region. The proposed solution is integrated with a Markov random field model to categorize event regions into separate homogenous regions. In this model, each sensor node contributes to detection procedure by providing local estimation the neighborhood information and its local observation. Performance analysis shows that this solution can detect the event regions accurately while minimizing the number of data transmissions required for accurate data reconstruction. A compressed network coding-based distributed data storage (CNCDS) method is proposed in [14]. It takes advantage of spatial correlation among sensor node measurements and aims primarily to achieve good recovery accuracy. Furthermore, it concentrates on reducing the number of data transmission and reception parameters, which results in energy-efficient data gathering. To do so, the proposed solution adjusts the probability of forwarding received data packets to the number of neighbor nodes. This approach ignores the noise and error parameters in the transmission scenario.

The research presented in [15] is one of the first studies to propose a compressive sensing-based data-gathering solution based on a random walk algorithm. The authors employ this algorithm to produce a non-uniform random measurement matrix. To do so, randomly selected sensor nodes in each walk generate a measurement based on a linear combination of both the node itself and neighboring nodes’ observations. At the end of each random walk, the measurements produced are forwarded to the base station. Compared to the existing random projection solutions, this approach shows a significant reduction in data-transmission cost.

Authors in [16] have mentioned that spatial-temporal correlation based compressive sensing solutions impose accuracy degradation in signal reconstruction procedure. To address this issue, they have proposed a weighted spatial-temporal compressive sensing solution that improves the data quality metric. However, it increases the number of data transmissions inside the network, which is still acceptable in the proposed solution.

A compressive sensing-based false data detection and correlation framework for crowd sourcing applications is presented in [17]. In crowd sourcing applications, a large number of participants employ their mobile devices to collect environmental data and report it to the base station. Misbehaving or erroneous participants can produce faulty data and packet loss in the network. Missing or faulty data is reconstructed with a spatial-temporal correlation-based compressive sensing technique. This approach considers collected data as multidimensional time-series and groups sensor nodes into smaller grids based on their location. In the first step, this solution gathers raw data from participants as a training data set and groups them into different grids by considering spatial correlation among sensor nodes. Then, the compressive sensing algorithm reconstructs the sensor data with spatial-temporal correlation and the low-rank feature among sensor node readings. The proposed solution guarantees high data quality in environments with high packet loss and noisy characteristics, but the study did not address energy savings.

A hybrid network coding and compressive sensing-based solution in [18] represents a clustered spatial-temporal correlation-based compressive sensing method. Each sensor node applies a well-defined Gaussian code matrix to encode its reading and forwards it to the base station. Proper selection of the measurement matrix and network coding coefficients is utilized at the base station to implement a low-complexity data reconstruction algorithm that reconstructs data more accurately. In addition, the proposed solution reduces the number of measurements, resulting in energy-efficient data transmission.

The authors of [19] propose a novel compressive sensing-based data-gathering solution for massive lossy data-transmission scenarios. The proposed compressive sensing solution models environmental data dynamicity using an environmental matrix (EM) and then employs the spatial-temporal correlation observed in this matrix to reconstruct the data. Furthermore, this approach has a multiple attribute assistant (MAA) component, which correlates environmental attributes to discover data loss patterns in reported encoded data. Combining MAA and EM, the compressive sensing-based reconstruction method provides better reconstruction accuracy. However, this approach suffers from higher computational complexity.

Another study [2] employs sequential data gathering and progressive data reconstruction to address a sliding window-based compressive sensing solution. Sensor nodes use this method to periodically encode and deliver their readings to the base station. The progressive data reconstruction method at the base station exploits Kronecker sparsification to recover the portion of sensor node readings reported during the last time period. This progressive method uses the recovered sensor readings from the last time period to restore encoded data in the current time period. There is a trade-off between data reconstruction accuracy and reconstruction complexity, which is adjusted by the sliding window size. In addition, the dynamic adjusting window size modifies the number of required measurements, which results in adaptive data transmission.

The authors in [20] address a two-step compressive sensing based data aggregation method in the clustered sensor network. Each cluster head utilizes a measurement matrix seed received from the base station to customize a localized measurement matrix and broadcast it into its cluster. Then, sensor nodes compress their measurements and forward it towards the cluster head. Upon receiving those readings, each cluster head aggregates and forwards their reading to the base station using an existing data routing tree among cluster heads. Simulation results show that the proposed solution improves network performance regardless of intra-cluster or inter-cluster on the total energy consumption of network.

## 3. Preliminarities

### 3.1. Compressive Sensing

This section presents the mathematical description of compressive sensing theory as presented in [21,22].

Let us assume that a discrete signal denoted by $X\in R^{N}$ represented by N×1 column vector has sparse representation in some basis (for example in a Fourier or wavelet basis). Considering the sparsity concept, this signal can be expressed in terms of its basis as follows:

(1) $$X=\Sigma_{k=1}^{N}a_{k}\psi_{k}=\Psi a,$$

where Ψ is an N×N orthogonal basis matrix $\Psi =[\psi_{1},\psi_{2},\ldots ,\psi_{N}]$, $\psi_{i}$, i=1,2,…,N and $a=[a_{1},a_{2},\ldots ,a_{N}]$ is an N×1 column vector of the coefficient sequence of *X* in Ψ domain.

Signal *X* is compressible or sparse in the Ψ basis if most of the elements in *a* are zero. Compressive sensing theory states that if signal *X* is K-sparse on Ψ basis, it can capture and recover from *M* non-adaptive, linear measurements (K<M<<N) with a certain restriction.

Compressive sensing theory also states that, rather than acquiring the entire signal and then doing compression, it should be possible to construct a sampling framework to capture only the useful information in the sparse signal to begin with. The sampled signal via compressive sensing is described as follows:

(2) Y=ΦX,

where $Y=[y_{1},y_{2},\ldots ,y_{M}]$ is the M×1 measurement matrix, $\Phi =[\phi_{1},\phi_{2},\ldots ,\phi_{M}]$ represents a M×N and each $\phi_{i}$, i=1,2,…,N is N×1 vector. It should be noted that Φ is a random matrix, which can be considered a second basis. Each elementary $y_{i}$ in the measurement matrix is a product of *X* and vector $\phi_{i}$ from the sensing matrix. We can replace *X* with $\Psi_{a}$ and rewrite *Y* as:

(3) Y=ΦX=ΦΨa=Θa,

where Θ=ΦΨ is a M×N matrix.

Compressive sensing demonstrates that sparse signals can be recovered from *M* measurements if the process can satisfy the restricted isometric property (RIP) [23,24]. The RIP states that the correlation between Φ and Ψ must be low (be incoherent) to restrict the number of measurements required to recover the signal. Coherence is a metric to measure the maximum correlation between any row of Φ and any column of Ψ and can be defined by the μ as follows:

(4) $$\mu \left(\Phi ,\Psi \right)=max_{1\leq k,j\leq N}\left|<\phi_{k},\psi_{j}>\right|.$$

When comparing Φ and Ψ, if the relationship between them is weak, the condition is called incoherence. Formally speaking, matrix Θ of size M×N satisfies the RIP of order *K* if it is a minimum number such that

(5) $$\left(1-\delta_{k}\right){∥a∥}_{2}^{2}\leq {∥\Theta a∥}_{2}^{2}\leq \left(1+\delta_{k}\right){∥a∥}_{2}^{2},$$

where $\delta_{k}\in \left(0,1\right)$ is a restricted isometric constant (RIC). Equation (3) must hold true for all values of *a* where a ${∥a∥}_{0}\leq K$ and ${∥a∥}_{0}$ is $l_{0}$ norm, which shows that the number of non-zero elements in *a*. A $l_{p}$ norm of vector *a* is defined as follows:

(6) $${∥a∥}_{p}^{p}=\Sigma_{i=1}^{N}{\left|a_{i}\right|}^{p}.$$

The RIP guarantees the exact recovery of *X* with a high probability if

(7) $$M\geq CK\log\frac{N}{K},$$

where *N* is the dimensionality of the original signal and *C* is the small positive constant. This formula is a conservative estimate of the lower bound on the number of measurements required to recover the original sparse signal. Since *K* represents the amount of important information inside the signal, we can conclude that the number of required samples to recover the signal is proportional to the information content of the signal.

The recovery of the signal *X* from *Y* is an NP hard problem, but it can be achieved through optimization. To do so, we can use $l_{0}$ minimization as follows:

(8) $$\overset{´}{a}=\arg\min_{a\in R^{N}}{∥a∥}_{l}{}_{0}\;s.t.\;Y=\Phi X,$$

where $\overset{´}{X}=\Psi \overset{´}{a}$, $\overset{´}{X}$ is the recovered signal and $\overset{´}{a}$ is the optimal estimation for *a*. Since $l_{0}$ minimization is computationally intractable, $l_{1}$ minimization is widely used for compressive sensing signal reconstruction. We can recover the coefficients of sparse signal *a* by solving $l_{1}$ norm minimization as follows:

(9) $$\overset{´}{a}=\arg\min_{a\in R^{N}}{∥a∥}_{l}{}_{1}\;s.t.\;Y=\Phi X.$$

### 3.2. Distributed Compressive Sensing

Standard compressive sensing methods can be implemented on a single sensor node. However, sensor networks consist of many sensor nodes distributed over the network. These sensor nodes have spatial and temporal correlation among their readings. Given this distribution, distributed compressive sensing is proposed as a standard compressive sensing method for several sensor nodes [25,26]. In this distribution, each sensor node employs compressive sensing theory to encode its reading and transfer it to the base station. For the reconstruction procedure, the base station utilizes joint sparse signal recovery to recover all encoded signals precisely.

Joint sparse signal (JSM) recovery involves a set of signals that are jointly sparse. Each joint sparse signal can be represented by a combination of the common part and the innovative part [27]. There are three different joint sparsity models that can be used to recover the original signals. In this case, we have an ensemble of signals with notation and measurement models as follows:

Each signal is denoted by $x_{j},j\in \left\{1,2,\ldots ,J\right\}$, where $x_{j}\in R^{N}$. For this model, it is assumed that there is a known sparse basis Ψ for $R^{N}$ in which each $x_{j}$ can be sparsely represented. On the other hand, each signal $x_{j}$ is assigned a measurement matrix Φ, which is $M_{j}\times N$. Based on this definition, a measurement signal for each signal *j* can be represented by $y_{j}=\phi_{j}\times x_{j}$.

In what follows, we introduce three different types of joint sparse models.

#### 3.2.1. Joint Sparse Model Type-1

The joint sparse model type-1, called JSM1, states that all signals reported by sensor nodes have a common sparse component while each individual signal contains an innovative sparse component:

(10) $$x_{j}=z_{c}+z_{j}\;with\;z_{c}=\Psi a_{c},\;{∥a∥}_{0}=K\;and\;z_{j}=\Psi a_{j},\;{∥a_{j}∥}_{0}=K_{j},$$

where the $z_{c}$ is common to the all of values for $x_{j}$ and its sparsity level is the minimum sparsity level of all the signals in base function Ψ. Signal $z_{j}$ is the specific innovative part of $x_{j}$ and has its sparsity level in the same base function. In this model, joint signal recovery maximizes reconstruction of the common part to increase the reconstruction accuracy. When the proportion of the common part is far more than the individual part, the reconstruction error decreases.

#### 3.2.2. Joint Sparse Model Type-2

The Joint sparse model type-2, namely JSM2, assumes that signals have the same sparsity set profile; however, their coefficients are different. In this model, we formulate each signal as:

(11) $$X_{j}=\Psi a_{j},$$

where $a_{j}$ is non-zero in the common coefficient profile. This models data for the sensor nodes that are deployed in the environment and observe the same signal, but, due to signal propagation effects, receive the signals with a phase shift.

#### 3.2.3. Joint Sparse Model Type-3

There is some similarity between Joint sparse model type-3 (JSM3) and JSM1, but the common profile is no longer sparse. As with JSM1, we formulate the signal as follows:

(12) $$x_{j}=z_{c}+z_{j}\;\;with\;z_{c}=\Psi a_{c},\;{∥a∥}_{0}=K\;and\;z_{j}=\Psi a_{j},\;{∥a_{j}∥}_{0}=K_{j}.$$

In this representation, $Z_{c}$ is not sparse. This model is applicable for environments in which several signals are produced from different sources with a background signal that is not sparse.

### 3.3. Sparse Transform Basis

Compressive sensing must utilize an appropriate transform basis to provide an accurate sparse representation for a compressible signal. The selection of these transform bases is very important and relates to the nature of the data or the signal to be compressed. In this section, we propose a classification for signals and then introduce an appropriate transform basis for each of them.

#### 3.3.1. Classification

A signal is a physical quantity that is measurable. Various classifications of signals have been suggested based on the nature of the signal and the system that produces it. These include continuous versus discrete, periodic versus non-periodic, energy versus power, deterministic versus random, stationary versus non-stationary, and linear versus nonlinear [28,29]. In our study, we classify existing signals into a linear and nonlinear signals. Linear here means that the natural frequencies of the signal are not dependent on the amplitude of oscillation. A signal can also be stationary or non-stationary. A stationary signal is one whose frequency or spectral contents change over time.

**Figure 1:**
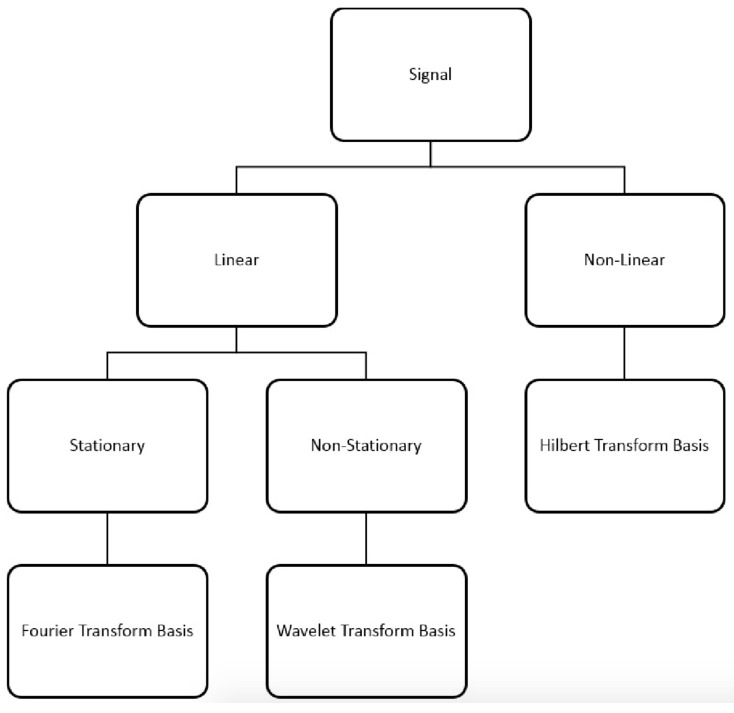
Signal classification and appropriate transform allocation.

The proposed signal classification and transform basis allocation are illustrated in Figure 1.

Based on the proposed signal classification, in this section, we introduce wavelet, Fourier and Hilbert transformations and discuss which transform basis belongs to which type of signal.

The Fourier transform is suitable for a wide range of purposes, such as signal transmission and stationary signal processing. However, this transform is not appropriate for non-stationary signals because it cannot provide any information about signal frequency changes as a function of time for these signals. To cope with this limitation, the short time Fourier transform (STFT) employs a fixed-size window to segment the signal into stationary parts but suffers from time or frequency resolution depending on its window size [28]. Most of the compressive sensing-based data-gathering approaches in wireless sensor networks use the Fast ourier transform (FFT) as their basis [30,31]. This basis implements transformation over the entire length of the signal. In signal reconstruction, this transform basis provides no information about time resolution and as such fails to report damage locations since all possible time information is lost. The STFT makes location information available. Although it provides a time-frequency representation of a signal, there is a major drawback with respect to utilizing STFT due to the fixed size of its window width [30,32].

The Wavelet Transform (WT) is a time-frequency technique that overcomes the poor time/frequency resolution and fixed-size window limitations of the STFT. In contrast to the FT, the WT is well localized, and, with it, few coefficients are needed to represent local transient structures. Therefore, the wavelet basis provides sparse representation of piecewise regular signals, which may include transients and singularities [29,33].

The variable window size employed by WT allows it to implement time-frequency transformation. When dealing with low- and high-frequency signals, it employs large windows and short windows, respectively (corresponding to long and short time intervals). Therefore, the accurate location of a transient signal can be determined, as well as fundamental frequency and its low-order harmonics [28].

The WT can be either continuous or discrete. The Discrete Wavelet Transform (DWT) is used for signal decomposition, while a Continuous Wavelet Transform (CWT) is used for spectral analysis [34]. Decomposing the CWT produces wavelet series. These series are highly redundant and their computation is costly and time-consuming. The DWT is a fast computation of WT that reduces time and resource costs [34]. Wavelet Multi Resolution (WMR) is a method that implements DWT with filters. It employs a high-pass filter to process rapidly changing details (high-frequency components) and a low-pass filter to find slowly changing features (low-frequency components). In contrast with the WMR, the Wavelet Packet Transform (WPT) is a method that decomposes both wavelet details and wavelet approximation components at each level. Wavelet packets inherit properties such as orthonormality and time-frequency localization from their corresponding wavelet functions [29,33].

There are number of basis functions that can be utilized as a mother wavelet for the wavelet transform, including Haar wavelet transform, Symlet wavelet transform, Coiflet wavelet transform, and Meyer wavelet transform. In terms of the signal characteristics and the attributes of specific wavelet transforms, each of them can be employed to represent sparsity of signal. The WT’s ability to provide good frequency resolution for high-frequency signals and time resolution for low-frequency signals makes this technique a good basis for non-stationary signals that require time and frequency resolution [29,34].

Hilbert transform is another transform basis that takes a function as an input and produces a function with the same domain. The Hilbert transform can be either continuous or discrete. The nonlinearity and non-stationary nature of some signals make the Hilbert transform a powerful tool as the transform basis for signals with those properties. Hilbert transform decomposes a signal posteriori, enabling the extraction of the inner scales of each signal [29].

### 3.4. Belief Propagation

**Figure 2:**
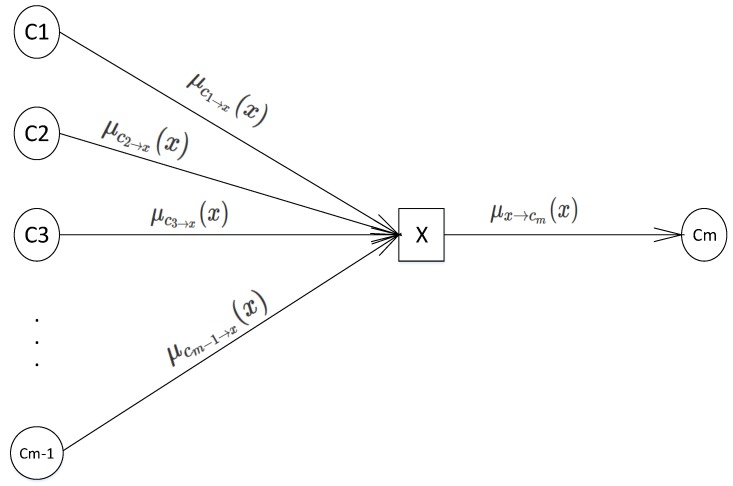
Message from variable node to the connection node.

**Figure 3:**
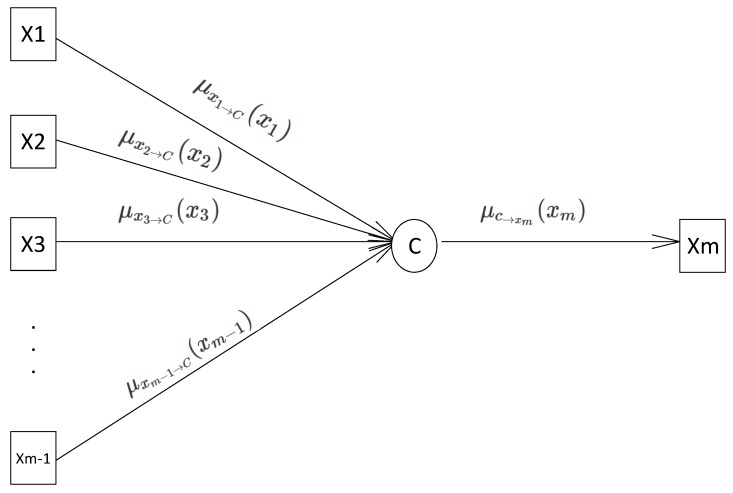
Message from connection node to variable node.

Belief propagation (BP) [35] is an iterative message passing algorithm that can calculate the marginal distribution or find estimates such as the most probable assignment (MAP) in Bayesian networks [36,37]. In the sparse signal recovery area, belief propagation is implemented on factor graphs and considered a fast decoder in Bayesian compressive sensing frameworks. This factor graph is a bipartite graph providing a visual representation of the sparse signal recovery procedure. It consists of two disjoint nodes: variable nodes and connection nodes connected through undirected edges whenever there is a dependency between these nodes. On the basis of belief propagation, these edges contain probability distribution functions on the variable nodes. There are only two directions for edges between variable and connection nodes [35]:

1. Edges or messages from a variable node to the connection node:
  These edges contain probability which is calculated by gathering all incoming edges (excepts edges coming from node $c_{m}$) and multiplying them, which is described as follows:
  (13) $$\mu_{x⟶c}\left(x\right)=\prod_{x\in N(x)\backslash \{c\}}\mu_{v⟶x}\left(x\right),$$
  where $\mu_{v⟶x}$ shows the edges from node *v* to node *x*, N(x) denotes the neighbor nodes of *x* and N(x)\{C} denotes the neighbor nodes of *x* except for node *C*. For an example, see Figure 2, which shows part of a factor graph. The message from variable nodes *X* to the connection node $c_{m}$ is given by:
  (14) $$\mu_{x⟶c_{m}}\left(x\right)=\mu_{c_{1}⟶x}\left(x\right)\times \mu_{c_{2}⟶x}\left(x\right)\times \ldots \times \mu_{(c_{m}-1)⟶x}\left(x\right).$$
2. Edges from a connection node to the variable node:
  This probability is computed by obtaining all the incoming edges to node *C* except the link from node *x*, multiplying them by *C* and finally finding the sum of all connected variable nodes except for node *x*. In general, the edges going from the connection nodes to the variable node can be described as follows:
  (15) $$\mu_{c⟶x}\left(x\right)=\sum_{∼\{x\}}c\left(N\left(c\right)\right)\prod_{v\in N(c)\backslash \{x\}}\mu_{v⟶c}\left(v\right),$$
  where N(C) is all variable nodes connected to *C* and σ is the sum of all connected variables except for *x*. $\mu_{v⟶c}\left(v\right)$ shows the edges from variable node *v* to the connection node *C*. An example is seen in Figure 3, which shows part of a factor graph. The message from connection nodes *C* to variable node $x_{m}$ is represented by
  (16) $$\begin{array}{c} \mu_{c⟶x_{m}}\left(x_{m}\right)=\sum_{\left(x_{1},x_{2},\ldots ,x_{m}-1\right)}c_{\left(x_{1},x_{2},\ldots ,x_{m}-1\right)}\times \mu_{x_{1}⟶c}\left(x_{1}\right)\times \mu_{x_{2}⟶c}\left(x_{2}\right)\times \\ \ldots \times \mu_{x_{m}⟶c}\left(x_{m}\right). \end{array}$$

### 3.5. Network Model and Assumptions

**Figure 4:**
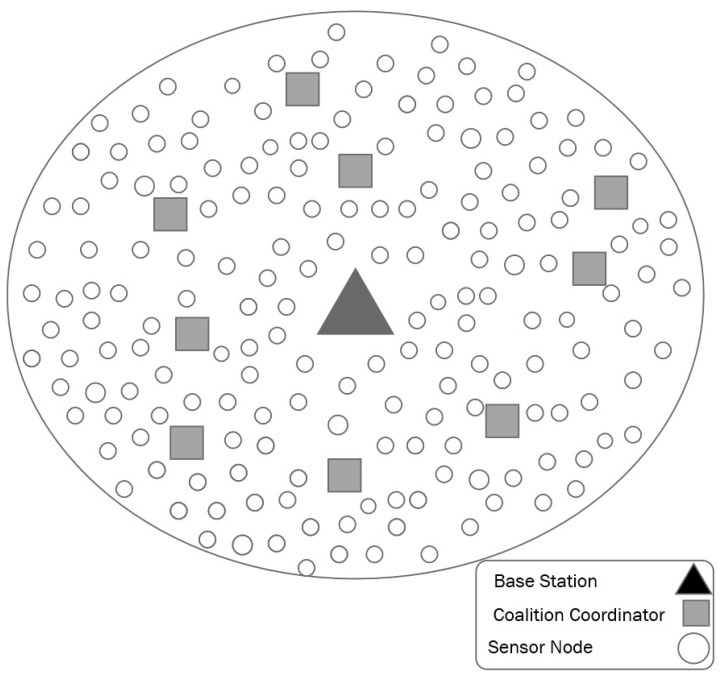
Network architecture.

Our heterogeneous network consists of *N* static sensor nodes regularly placed inside a region as shown in Figure 4. It utilizes multi-hop communication to forward the readings to the base station. Each sensor node is assigned an integer ID, *n*, which is in the range from 1 to *N*.

There are three types of sensor nodes in the network: normal nodes, coalition coordinator and base station. The normal sensor nodes have limited computational and storage capacity. Moreover, these sensor nodes are equipped with limited battery power, as recharging or replacing their batteries is difficult and expensive. There are a few powerful but also resource-restricted nodes: the coalition coordinators, which have more memory and computation capabilities. The base station is a powerful node with significant computational and storage resources. The network follows a periodic monitoring scenario to gather data from the environment. In such a long-term monitoring application, the end user requires a continuous stream of environmental data, although nothing specific is being detected. In these sorts of applications, energy consumption and data accuracy requirements are more important than real-timeness.

**Figure 5:**
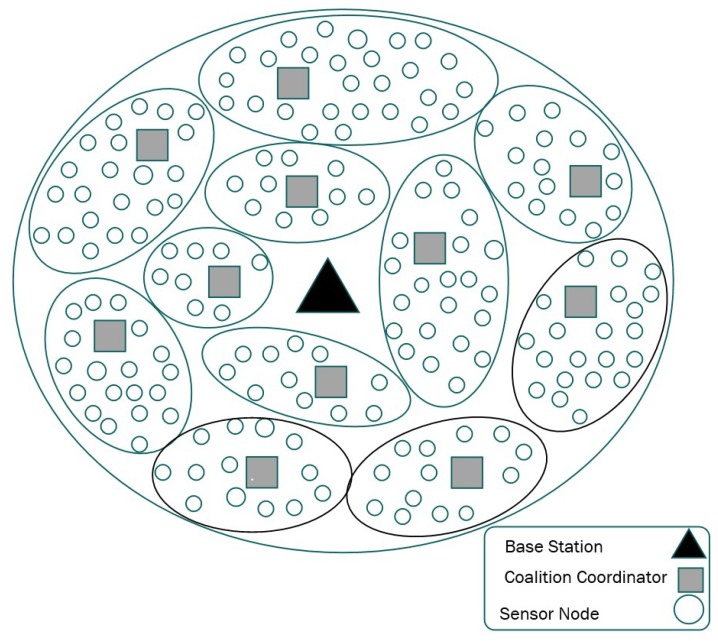
Coalition representation.

The sensor nodes are grouped into coalitions. To form these coalitions, we consider the sparsity distribution of signals among sensor nodes and place all correlated sensor nodes in the same group (Figure 5).

## 4. Overview of the Proposed Approach

In order to achieve an energy-efficient and quality-aware compressive sensing method, we introduce a distributed compressive sensing approach with spatial correlation among sensor nodes to group them into coalitions. The proposed coalition-formation method is represented by a block diagonal measurement matrix in which each diagonal entity corresponds to one of the coalitions. Upon forming coalitions, the proposed spatial-temporal correlation-based compressive sensing approach is implemented inside each coalition in order to schedule sensor nodes and encode their readings. Temporal correlation among sensor node readings allows our approach to adjust the number of measurements with regard to the temporally changing sparsity level.

After applying the proposed compressive sensing solution inside each coalition and forwarding compressed data, the base station employs a two-step joint sparsity-based recovery algorithm to reconstruct the original signal. In the first step, it runs a joint sparsity model to find the common sparsity profile among the coalitions. In the second step, it calculates the common sparsity model within each coalition, which allows it to achieve data reconstruction with high accuracy and fewer measurements.

We summarize our contributions in this paper as follows:

- Proposing a new compressive sensing-based coalition-formation mechanism that includes distributed compressive sensing-based data gathering. This mechanism balances energy and quality of service.
- Introducing a sparsity distribution-based sensor node scheduling technique that minimizes the number of active sensor nodes.
- Proposing an adaptive measurement mechanism that adjusts the number of measurements inside each coalition according to the temporal sparsity level of the signal.
- Proposing a two-level belief propagation-based reconstruction algorithm that provides acceptable data quality in terms of data accuracy.

**Figure 6:**
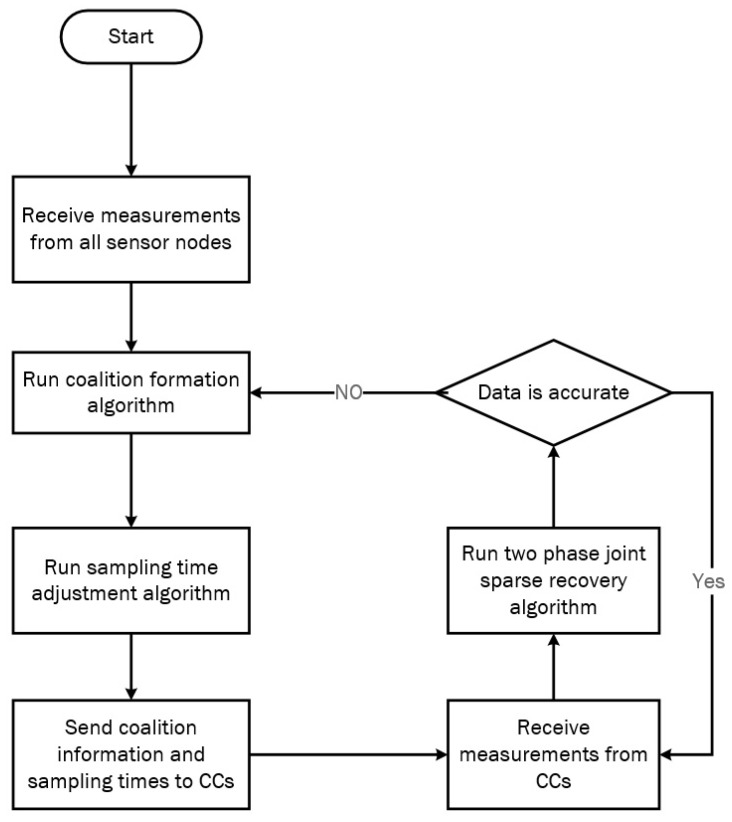
Approach overview flowchart.

For a graphical view of the entire data-gathering procedure proposed in this paper, see the flowchart in Figure 6. This flowchart will be described in more detail in the following sections.

## 5. Coalition Formation

We utilize spatial correlation among sensor nodes and a base function distribution pattern in order to group sensor nodes into several coalitions. Using coalitions leads to an energy-efficient compressive sensing-based measurement system that puts correlated sensor nodes close to each other in the same coalition. Moreover, coalitions make our measurement system sparse, which means each measurement is obtained through a linear combination of samples captured by a few sensors within a single coalition. Using this coalition structure, our approach decreases transmission cost. In addition, within each coalition, we remove the redundancy among compressed measurements of different sensors, decreasing the number of data packets.

The compressive sensing-based coalition formation introduced here is inspired by the fact that the subject signals are distributed in the environment. On the other hand, the distribution of the signal elements is different for the sensor nodes based on their locations. Therefore, the sensor nodes observe the same signal with different resolution. On the other hand, the sparse representation of the signal can be shown in a sparsity base function. We apply the base function distribution over the network to define a cover parameter. This cover metric shows the degree of the base function covered by the sensor nodes. This parameter helps sensor nodes inside each coalition produce informative measurements, which results in accurate data recovery by the base station.

To make a concrete and accurate coalition, we define a utility function *U* based on cover, transmission and sensor node correlations. This function is used to evaluate the efficiency of the coalitions produced. To do so, *U* makes a trade-off between reconstruction accuracy (correlation between sensor nodes and base function distribution pattern) and data-transmission cost (distance between sensor nodes).

The following sections explain the prerequisites for the coalition-formation algorithm and conclude by presenting its procedure.

For the sake of readability, we have summerized a list of notations used in the following sections in Table 1.

### 5.1. Measurement Matrix

Since we divide the network into different coalitions, the data is gathered through these different coalitions. Therefore, whole data may be divided into discrete blocks, in which each block is acquired via a local measurement operator. Assume that we have divided the network into $N_{C}$ coalitions and our signal *X* is partitioned into $N_{C}$ blocks $X_{1},X_{2},\ldots ,X_{N_{C}}\in R^{N}$. Each block *j* which shows coalition *j* is assigned with a local measurement sub-matrix $\Phi^{j}:R^{N}\to R^{N_{j}}$. Each measurement sub-matrix $\Phi^{j}$ represents the measurement pattern in each coalition *j*.

Each of our measurement sub-matrixes $\Phi^{j}$ is assigned to one coalition matrix *C*, which permutates the entities of the original signal assigned for the specific coalition j. In order to assign each coalition *j*, through its measurement matrix $\Phi^{j}$ with the permutated signal coefficients $x_{j}^{C}$, we multiply *C* with *X*, which produces $CX=X^{C}=\left[x_{1}^{C},x_{2}^{C},\ldots ,x_{N_{C}}^{C}\right]$ and finally we have:

(17) Y=ΦCX,

where:

(18) $$\Phi =\left(\begin{array}{ccccc} \Phi^{1} & & & & \\ & \Phi^{2} & & 0 & \\ & & . & & \\ & 0 & & . & \\ & & & & \Phi^{N_{C}} \end{array}\right).$$

The resulting matrix ΦC represents the distribution of sensor nodes inside each coalition and *C* is a coalition matrix.

Based on Equation (18) and sparse representation of the signal $X=\tilde{\Psi }a$, we obtain:

(19) $$Y=\Phi CX=\Phi (C\tilde{\Psi })a=\Phi \Psi a,$$

where $\Psi =C\tilde{\Psi }$ is a permutated version of $\tilde{\Psi }$. As we have seen, our coalition matrix permutates the base functions. Through this permutation, each row in Ψ is a permutated row of $\tilde{\Psi }$ as follows:

(20) $$\psi_{i}^{T}=\sum_{j=1}^{N}P\left(i,j\right){\tilde{\psi }}_{j}^{T},$$

where $\psi_{i}^{T}$ and ${\tilde{\psi }}_{j}^{T}$ are the row vectors of Ψ and $\tilde{\Psi }$, respectively. If P(i,j)=1, then the $i^{th}$ row of Ψ is replaced with $j^{th}$ row of $\tilde{\Psi }$.

After permutation with the coalition matrix, we have a proper pattern for each $\Phi^{j}$ to measure data from the sensor node inside each coalition *j*.

The coalition-formation mechanism proposed here leads to a block diagonal measurement matrix with the appropriate coalition matrix *C* relative to the location of the sensor nodes.

### 5.2. Utility Function

The proposed coalition matrix *C* can be assigned to any coalition-formation method. During the coalition-formation procedure, we aim to make the best trade-off between energy savings and reconstruction accuracy. To do so, we introduce a utility function to evaluate the efficiency of the coalition-formation process. This utility function is defined based on the energy, correlation and cover degree parameters. The following sections explain these parameters in more detail and conclude by introducing the utility function.

#### 5.2.1. Energy

We define the energy parameter for coalition-formation scenarios based on transmission, processing and measurement costs. Energy consumption is described as follows:

(21) $$E_{i}=E_{comm}+E_{pro}+E_{meas},$$

where $E_{comm},E_{pro},E_{meas}$ are communication, processing and measurement energy parameters, respectively. In this section, we focus on data transmission and measurement cost. We normalize these costs and replace energy requirements for the processing and measurement with the measurement costs. The transmission cost relates directly to the distance, while the measurement cost is influenced by the number of measurements. We replace the energy parameter with the normalized parameters of distance between two nodes inside the coalition and the number of measurements is represented as follows:

(22) $$ECo\left(i,j\right)=\frac{Dist(i,j)}{Dist_{max}}+\frac{M_{i}}{M_{max}},$$

where $ECo_{i}$ is the energy cost in terms of the normalized distance between nodes *i*,*j* Dist(i,j) and the normalized number of measurements taken by node *i* ($M_{i}$).

#### 5.2.2. Correlation Degree

Since sensor nodes are located close to each other and they sense the same signal with different resolutions, we are able to find different levels of spatial correlation among them. Existing compressive techniques usually ignore this correlation and transfer redundant compressed data, which costs energy. Our approach tries to remove the redundancy among compressed data, thus leveraging this spatial correlation. To do so, in the coalition-formation phase, we force the algorithm to consider this correlation along with other parameters. When we have correlated sensors in the same coalitions, we remove redundancy in the compressed data using the algorithm described in the next section. The correlation metric among sensor nodes is defined as Corr:

(23) $$Corr\left(i,j\right)=\frac{Cov(y_{i},y_{j})}{\sigma \left(y_{i}\right)\sigma \left(y_{j}\right)}.$$

According to this formula, we define a binary variable CR which indicates whether two sensor nodes are sufficiently correlated. For this purpose, we rely on a user-defined correlation threshold TH1:

$$CR\left(i,j\right)=\left\{\begin{array}{cc} 1\; & if\;\;\;Corr(i,j)>TH1,\\ 0\; & if\;\;\;Corr(i,j)\leq TH1. \end{array}\right.$$

#### 5.2.3. Cover Degree

Measured signals can be represented by a sparsity function distribution over the networks. These functions can also be grouped into one or more coalitions. The performance of our coalition mechanism in terms of recovered data accuracy greatly depends on the nature of the sparsity base functions. To evaluate this performance, we define a sparsity base cover degree (SCD) metric, which measures the degree of overlap between each coalition with base functions Ψ. Essentially, the SCD shows the energy overlap between the base functions and coalitions.

We define the SCD metric between each base function *i* and coalition $Col_{j}$ as follows:

(24) $$SCD\left(j,i\right)=\sum_{m\in Col_{j}}\psi^{2}\left(i,m\right),$$

where *m* is a sensor node located in coalition *j*. This metric SCD(j,i) indicates that measurements collected from coalition *j* contain information about the measurements of other coalitions that cover the same base function *i*. Taking this coverage degree among different coalitions into account, we use a joint sparse signal recovery approach to recover the original signal. However, there are situations in which Ψ is covered only by one coalition. This means that, for a K-sparse signal, the sparsity bases are contained in one coalition. Since this coalition is not known beforehand, we need to gather data from all coalitions, which is not energy efficient. Meanwhile, for accurate data recovery, we must have O(KlogN) measurements, meaning that we need $O(KN_{C}\log N$) measurements from all coalitions. On the other hand, the redundant measurements from other coalitions that do not overlap with the base do not contribute to improving data accuracy. If Ψ even has overlap among different coalitions, our data recovery accuracy is increased. To quantify the cover level of Ψ over coalitions, we define the maximum SCD as follows:

(25) $$SCD_{max}\left(\Psi \right)=SCD_{max}\left(C\bar{\Psi }\right)=max_{j,u}\sum_{w}\Psi^{j^{2}}\left(w,u\right),\;SCD\left(\Psi \right)\in \left[0,1\right].$$

$SCD_{max}$ shows the maximum cover level of each coalition with the sparsity base, while $\Psi^{j}$ is the base sub-matrix assigned to coalition *j*.

Forming coalitions such that the sparsity function can be recovered with several coalitions increases the quality of the recovered data. On the other hand, we must lower the number of correlated coalitions and the number of measurements to cut energy consumption. To find this balance, we must define a utility function as described in the next section. We discuss the number of coalitions and measurements and how $SCD_{max}$ makes a trade-off between energy and quality in terms of the minimum number of measurements and data recovery accuracy.

#### 5.2.4. Utility Function Formulation

In this section, we calculate a utility function *U* to evaluate the candidate coalition structures.

In the coalition-formation phase, we aim to build $N_{C}$ coalitions to achieve minimum energy consumption while meeting data-quality requirements. Minimizing transmission and measurement costs, which is the main energy consumption parameter here, depends on $SCD_{max}$, $M_{i}$ and $Dist_{ij}$.

The proposed utility function should evaluate adding a new sensor node to any coalition in terms of the trade-off between energy and quality. To do so, we define the utility function *U* for each combination of $\left(n_{i},Col_{r}\right)$ as follows:

(26) $$U\left(n_{i},Col_{r}\right)=CR\left(n_{i}\right)\times \left(ECo\left(n_{i},Col_{r}\right)+\alpha SCD_{max}\left(n_{i},Col_{r}\right)\right),\;\alpha >0,$$

where $CR(n_{i})$ defines the correlation level of sensor node *i* with other sensor nodes in $Col_{r}$. Regarding the $SCD_{max}\left(n_{i},Col_{r}\right)$ parameter, adding a sensor node to different coalitions may change the value of $SCD_{max}$.

### 5.3. Coalition Formation Algorithm

In the model introduced in this paper, the network consists of *N* sensor nodes $SN=\{n_{1},n_{2},\ldots ,n_{N}\}$ and $L=\{l_{n_{i},n_{j}}\}$ is the set of all possible connections between sensor nodes. Two sensor nodes are considered to be connected if they are placed in communication range of each other. We assume that Ψ is known and all sparsity bases are normalized to 1 so that SCD∈[0,1]. This algorithm aims to cut energy consumption, provided that the data-quality requirement is met. Adding a new sensor node to the coalition is a selection procedure that evaluates which coalition is the best candidate for adding a sensor node. To make a good selection, we run an optimization algorithm on *U*.

Before describing the optimization algorithm, the SCD parameter must be redefined. During coalition formation, when we add a new sensor node to the coalition, SCD evaluates the effect of adding this node by assigning a weight for the link between existing node in the coalition and the new sensor node. Our coalition-formation method, in order to examine the effect of adding a sensor node to each coalition, defines the SCD factor as $SCD_{max}\left(l_{n_{i},n_{j}},Col_{r}\right)$ by considering the link, $l_{n_{i},n_{j}}\in L$ and a given coalition $Col_{r}$. This link does not connect two nodes in the same coalition, which means $\;n_{i}\in Col_{r}\;\;and\;\;n_{j}∋Col_{r}$.

Now, we redefine the utility function *U* for each combination of $\left(l_{n_{i},n_{j}},Col_{r}\right)$:

(27) $$U\left(n_{i},Col_{r}\right)=CR\left(n_{i},n_{j}\right)\times \left(ECo\left(l_{n_{i},n_{j}}\right)+\alpha SCD_{max}\left(l_{n_{i},n_{j}},Col_{r}\right)\right).\;\alpha >0$$

$SCD_{max}\left(l_{n_{i},n_{j}},Col_{r}\right)$ here represents the maximum cover level when a new node is added to coalition $Col_{r}$ using the link $l_{n_{i},n_{j}}$.

Now, we can formalize our optimization algorithm. The main goal in the improvement procedure is to lessen energy consumption and data recovery errors by adding new sensor nodes to the coalitions. When selecting an appropriate coalition for a new sensor node, the optimization algorithm examines the utility of adding the new sensor node to each coalition. For each coalition, this utility is defined based on the link connecting the new sensor node to an existing node in the coalition. Running the optimization algorithm finds the coalitions that pare down utility function’s link cost. We formulate this optimization as follows:

(28) $$\begin{array}{c} \left(n_{i},l_{min},Col_{r_{min}}\right)=\arg\min U\left(n_{i},Col_{r}\right)=\arg\min[CR\left(n_{i},n_{j}\right)\times \\ \left(ECo\left(l_{n_{i},n_{j}}\right)+\alpha SCD_{max}\left(l_{n_{i},n_{j}},Col_{r}\right))\right]\\ subject\;\;\;to\;\;\;n_{i}\in SN,\;n_{j}\in Col_{r},\;CR\left(n_{i},n_{j}\right)\in \left\{0,1\right\}. \end{array}$$

Now let us describe the algorithm according to the optimization equations. To find a set of sensor nodes for each coalition, this algorithm uses Equation (28) to find a set of links such that the total *U* of the links is minimized. In the initialization step, we assume a set of candidate nodes and candidate links to be added to the coalitions defined by a set of SN and *L*. The algorithm then assigns each coalition coordinator node CC to one of the $N_{C}$ coalitions. In addition, it defines $SN_{Col_{r}}$ and $L_{Col_{r}}$ as a set of sensor nodes and connections of coalition *r*, respectively. Along with the initialization step, the algorithm runs an iterative procedure where, in each iteration, it allocates one sensor node to one of the coalitions. To do so, it first finds the utility function for all possible connections defined in *L*. It then runs the optimization function defined in Equation (28) and finds the minimum utility. However, it finds the utility for sensor nodes that satisfies the minimum correlation requirements. The output of this optimization is a connection link with minimum utility $l_{min}$. This link connecting $\left(n_{i},n_{j}\right)$ adds sensor node $n_{i}$ to the coalition of which node $n_{j}$ is a member. Upon adding a new sensor node, the SCD parameter of the all links connected to the coalitions changes so that the link utility of the sensor nodes may change. It then removes this link and sensor node $n_{i}$ from the list of candidate links and nodes. This procedure continues until all sensor nodes are assigned to the coalitions. Algorithm 1 represents this coalition formation procedure.

**Algorithm 1** Coalition formation
1:
$SN=n_{1},n_{2},\ldots ,n_{N}$
2:Define $L=l_{ij}$ as a set of all possible links3:Define $N_{C}$ coalitions with one coalition coordinator4:Define set of nodes $N_{col_{k}}$ and links for each coalitions $L_{col_{k}}$5:
**for** $\left(P=1;P\leq \left(N-N_{C}\right);P++\right)$ **do**
6: **for** (Q=1;Q≤|L|;Q++) **do**7:  $Find\;\;L_{poss}=l_{ij};\;i\in N_{col_{k}},j\in SN$8: **end for**9: Cacluate $U(I_{ij},Col_{k})$10: Add $n_{j}$ to $Col_{k_{min}}$11: Add $l_{min}$ to $L_{Col_{min}}$12: Find $\left(n_{j},l_{min},Col_{k_{min}}\right)$13: Remove $n_{j}$ from SN14: Remove $l_{min}$ from *L*15:
**end for**



## 6. Data Gathering inside Coalitions

For the sensor nodes inside each coalition, we use a distributed compressive sensing technique to take measurements and recover the original signal. Before describing the data-gathering approach, in this section, we take advantage of the SCD parameter to find the minimum number of active sensor nodes inside each coalition. The active sensor nodes inside the coalition send their reading through a multi-hop network structure to the coalition coordinator.

### 6.1. Number of Active Sensor Nodes

In this section, we identify the number of sensor nodes required to be active for each coalition. This requirement helps to implement scheduling among sensor nodes and put some of the sensor nodes into sleep mode. Since the number of active sensor nodes measuring the signal is in direct relation with the number of measurements, we use Equations (4) and (7) to find the number of active sensor nodes.

To do so, we first define a minimum threshold based on Equation (4) to measure the minimum data reconstruction quality. We then measure the probability of having coherence, as defined in Equation (4), higher than this threshold to make sure that the number of sensor nodes is enough to recover a compressed signal with high accuracy. This probability is defined and represented as follows:

If all measurement matrixes $\phi_{j}$ are orthogonal and the sparsifying basis function $\tilde{\Psi }$ and coalition-formation matrix *C* are known a priori, then μ(θ) is bounded as follows:

(29) $$Pr[\mu \left(\theta \right)\leq O\sqrt{SCD_{max}\frac{N_{C}}{N}\log N}\;]=1-O\left(\frac{1}{N}\right).$$

Considering Equations (7) and (29), we can define the maximum number of sensor nodes required for providing high quality data as follows:

(30) $$N_{ANodes}=O\left(SCD_{max}KN_{C}\log^{2}N\right).$$

Fine-tuning the coalition-formation matrix leads to a smaller $SCD_{max}$, which in turn produces a lower number of sensor nodes while still ensuring data quality. The number of active sensor nodes associated with each coalition is dependent on its $SCD_{max}$ level. Coalitions, which cover more sparsity functions, are more informative. Therefore, we must gather more data from those coalitions.

The number of active sensor nodes for each coalition is represented as follows:

(31) $$P_{j}=N_{Col_{j}}=\frac{SCD_{Col_{j}}}{SCD_{max}}N_{ANodes}.$$

### 6.2. Compressive Sensing Based Data Gathering

We now construct a block diagonal measurement matrix using the spatial-temporal correlation among sensor nodes.

Let $SN_{Col_{j}}=\left\{1,2,\ldots ,N_{j}\right\}$ denote the set of sensor nodes for the jth coalition, where $P_{j}$ of these sensor nodes are randomly scheduled to be active. In contrast to existing compressive sensing methods, we define a new structure for the measurement matrix that is compatible with our coalition-formation method. We use a temporal block diagonal measurement matrix, $\Phi_{t}$, to gather data. During each sampling instance, we gather spatial observations of all sensor nodes at time *t* and produce a discrete spatial signal $X_{t}$ at this time. The temporal observation of all active sensor nodes together is a spatial-temporal signal $\left[X_{1}^{tr},X_{2}^{tr},\ldots ,X_{ST}^{tr}\right]$, where ST is a parameter representing the number of samples in each sampling round *T*. Each sampling period consists of *T* sampling instances equal to the Shannon–Nyquist rate. To reduce this number of sampling times, the base station adjusts the number of sampling times according to the signal sparsity level and defines a number of sampling points ST for each sampling period.

For each sampling period *t*, we consider $\Phi_{t}$ as a measurement matrix; $\Phi_{t}$ is a $P_{j}\times SN_{Col_{j}}$ matrix. The measurement vector $Y_{Col_{j}}$ consists of $ST_{j}$ sub-vector of ST sampling times such that $Y^{Col_{j}}=\left[Y_{1}^{tr},Y_{2}^{tr},\ldots ,Y_{ST_{j}}^{tr}\right]$ where each $Y_{i}$ is a $P_{j}\times 1$ vector.

Inside each coalition, we utilize a block diagram measurement matrix that compactly represents several temporal measurement sub-matrices. Combining these spatial-temporal measurements together yields:

(32) $$Y^{j}=Y_{Col_{j}}=\Phi^{j}X^{j},$$

(33) $$Y^{J}=\left[\begin{array}{c} Y_{1}\\ Y_{2}\\ .\\ .\\ .\\ Y_{ST_{j}} \end{array}\right]=\left(\begin{array}{ccccc} \Phi_{1} & & & & \\ & \Phi_{2} & & 0 & \\ & & . & & \\ & 0 & & . & \\ & & & & \Phi_{ST_{j}} \end{array}\right)\left[\begin{array}{c} X_{1}\\ X_{2}\\ .\\ .\\ .\\ X_{ST_{j}} \end{array}\right],$$

where for each 1≤t≤ST, $\Phi_{t}$ has $P_{j}$ rows and $SN_{Col_{j}}$ columns. At the end of each sampling round, every sensor node transfers its measurement vector $Y_{j}$ to its neighbor node. The neighbor node receives the data and forwards it to the coalition coordinator.

**Figure 7:**
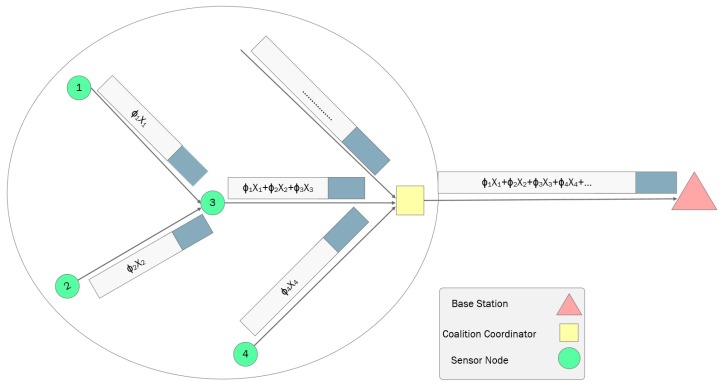
An example gathering scenario.

Introducing this temporal block diagonal measurement inside each coalition provides energy-balanced data aggregation within the coalitions. For instance, consider the sample coalition in Figure 7, where active sensor nodes forward their compressed data through active neighbor nodes to the coalition coordinator. Existing compressive sensing-based data collection approaches introduce unbalanced data transmission, which leads to communication and data overhead in the network. In our approach, each sensor node transfers a total of *M* number of measurements where $M_{j}=m_{1}+m_{2}+\ldots +M_{SR_{J}}$. For each sampling time *t*, sensor node *i* sends its measurement $y_{i},t$ to the next node *j*.

For each time *t*, we represent the data-gathering process as follows:

(34) $$y^{t}=\Phi^{t}x^{t},$$

where $\Phi^{t}=\left[\phi_{1},\phi_{2},\ldots ,\phi_{N_{Col_{k}}}\right]$ is the measurement matrix of size $P_{Col_{k}}\times N_{Col_{k}}$ and $y^{t}$ is the compressed measurements of signal $x^{t}$.

Each element $y_{i}$ of measurement vector $y^{t}$ is a linear combination of all sensor node readings inside the coalition, which is weighted with a row of the measurement matrix. This representation of each measurement gathered from active sensor nodes at time *t* can be represented as follows:

(35) $$y_{i}^{t}=\sum_{j=1}^{N_{Col_{k}}}\phi_{ij}^{t}x_{j}^{t},\;i\in 1,2,\ldots ,P_{Col_{k}}.$$

Using this representation, we balance energy usage through an equal number of transmissions among sensor nodes inside each coalition. According to the proposed temporal representation, each sensor node is able to add its measurements ${\left\{\phi_{ij}^{t}\right\}}_{i=1}^{P_{Col_{k}}}x_{j}^{t}=\phi_{j}^{t}x_{j}^{t}$ to the measurements of other sensor nodes separately to produce each measurement. To do so, each $P_{Col_{j}}$ active sensor node inside the coalition locally multiplies its readings by measurement vector $\phi_{j}^{t}$ and produces its measurement vector $M^{t}$. It then waits until it receives measurement vectors from other sensor nodes that forward their data to the voalition coordinator (CC) through this sensor node. Upon receiving them, this sensor node aggregates its measurement vector with the received measurement vector and forwards it to the CC. This procedure is depicted in Figure 7.

One question not yet answered concerns the means of routing sensor node measurements to the coalition coordinator. In the next section, we describe how sensor nodes form a routing tree and forward their measurements.

### 6.3. Data Gathering Trees

Inside each coalition, our network consists of $P_{j}$ active nodes connected through a set of links *L*. For each time stamp, every sensor node reads environmental data and transmits it to the coalition centre. Since not all sensor nodes have access to the coalition centre, they forward their readings with multi-hop communication. In the compressive sensing technique, each coalition centre requires gathering only *m* projections from these sensors instead of all readings from sensor nodes where m<<n.

As indicated above, each measurement is the weighted sum of active sensor node readings with non-zero coefficients. Our temporal measurement matrix consists of $m_{j}$ rows and $P_{j}$ columns where each column is assigned for one active sensor node and each row shows one of the projections. Since each row shows the selected sensor nodes whose readings contributed to the weighted sum $m_{j}$, we can consider each row as a data-gathering tree. In this regard, we define one of the sensor nodes with direct access to the coalition centre as a root of this tree. Therefore, having $m_{j}$ projection, we obtain $m_{j}$ data-gathering tree. To distribute the non-zero coefficients more evenly in matrix ϕ and make each projection as sparse as possible, the number of non-zeros in each row of matrix ϕ is set at $\left\lceil \frac{m_{j}}{P_{j}}\right\rceil$ such that none of the columns in ϕ has all-zero entries.

We now try to find $m_{j}$ data-gathering trees, each of which corresponds to one weighted sum of selected sensor nodes such that it minimizes the energy consumption by minimizing communication links. The following section describes how each projection node builds its measurement tree.

#### 6.3.1. Building Data Gathering Tree

Selecting $m_{j}$ projection nodes and assigning a data-gathering tree based on the optimized random basis matrix are the main objectives of this section. To select $m_{j}$ projection nodes, the coalition coordinator randomly selects $m_{j}$ nodes that are one hop from it, and fixes them as roots of *m* projection trees. Later, during the data-gathering procedure, the coalition coordinator can change the role of projection nodes and notify these nodes by sending update messages.

Each projection node initializes one of $m_{j}$ data projection procedures using the proposed algorithm. As mentioned above, to build the data-gathering tree for each projection, we construct an optimized basis matrix and assign each row of this matrix to one of the projection trees. Each column of this projection matrix is assigned for one of the active sensor nodes, while each row is assigned to one projection node. Non-zero coefficients in each row represent the nodes that participate in that projection.

To make each random projection as sparse as possible, this algorithm sets the number of non-zero entities in each row of the measurement matrix ϕ as equal to $\frac{m_{j}}{P_{j}}$, on the condition that none of the columns in ϕ has all-zero entries. The proposed measurement matrix has been generated in advance and stored in each sensor node. Now, we demonstrate building data projection trees within each coalition.

In each coalition, the coalition coordinator gathers $m_{j}$ weighted measurements from all active sensor nodes inside the coalition. Each of these $m_{j}$ measurements is collected by one of the measurement nodes. To do so, each row of our measurement matrix represents the data-gathering tree assigned to one measurement node and each binary entity in this row vector shows whether this node is participating in the current measurement. Each measurement node uses this measurement matrix to detect sensor nodes that will participate in the measurement. It then builds a data-gathering tree based on the minimum spanning tree algorithm to develop a route between participating nodes and the measurement node.

The measurement node initiates the data-gathering tree by introducing itself as a root. It then finds all participating nodes located within one hop and adds them to the tree. For other nodes, it finds the closest node and follows the shortest path method to connect that node to the tree. This procedure repeats until all participating nodes join the tree. When the tree is complete, each sensor node takes its sample $x_{i}$, multiplies it by its coefficients, adds it to the data received from its child nodes and finally transmits the weighted sum to its parent node. At the end of this procedure, the measurement node receives all the weighted measurements from the tree and forwards them to the base station. Since the measurement matrix consists of $m_{j}$ rows, we will have $m_{j}$ trees, each of which gathers one of the measurements through the assigned measurement node.

Upon reading a sample from the environment, each sensor node in each tree multiplies its reading by its coefficient, adds it to the data received from its child nodes and sends the weighted sum to the parent node. Repetition of this procedure in the tree terminates when the measurement node receives weighted sums from the child nodes and then sends these readings to the coalition coordinator. In this way, all sensor nodes transmit only one packet to the coalition coordinator. Consequently, for *m* data-gathering trees, the network transmits *m* packets.

## 7. Joint Sparse Signal Recovery Procedure

The base station is responsible for recovering the original signals from the measurements received from the coalitions. Considering several coalitions, signals gathered by the coalition coordinators are correlated in spatial and temporal domains. In essence, we have two types of correlations: spatial-temporal correlation inside each coalition and spatial correlation among coalitions. The distributed sparse signal approach proposed here engages these correlations and suggests a two-step joint sparse signal recovery process. In the joint sparse signal recovery concept [38,39], this correlation can be defined based on location or amplitude of non-zero coefficients of the signal. In our approach, correlation among different coalitions or sensor nodes is depicted as a similarity level and is defined based on the location of the non-zero coefficients as follows:

(36) $$SL=\frac{Com}{K},$$

where Com and *K*, respectively, are the total number of common non-zero coefficient locations among different coalitions or sensor nodes and the total number of non-zero coefficients of the signals (sparsity level).

At the end of each data reconstruction procedure, the base station calculates and finds the common sparsity profile $CSP_{Col}$ among different coalitions. Coalitions that satisfy minimum similarity requirements are considered in the joint sparse signal recovery process; otherwise, their recovery proceeds separately.

Based on this SL definition, in this section, we develop a two-step joint sparse signal recovery algorithm which uses the spatial-temporal prior information to reconstruct the original signal. Using this algorithm, our approach decreases the number of measurements while increasing the reconstruction accuracy.

Our signal recovery procedure is performed both inside the coalitions and among the coalitions. In the first step, the base station runs the joint sparse signal recovery algorithm among coalitions and finds their common sparsity profile ($CSP_{Col}$). In the second step, using $CSP_{Col}$ as an input for the reconstruction algorithm, it runs joint sparse recovery inside each coalition to complete the common sparsity profile of each coalition ($CSP_{Col_{j}}$). Since the sensor nodes inside each coalition are more highly correlated, running the second round yields other parameters of the common sparsity profile. Upon finding the common profile among sensor nodes ($CSP_{Col_{j}}$), the base station runs the individual recovery procedure to find individual parts. For the whole recovery process, the base station exploits the belief-propagation-based recovery algorithm introduced in our previous work [40]. Algorithm 2 summarizes the proposed recovery procedure.

**Algorithm 2** Two-step joint signal recovery
1:Find SL among different coalitions2:Find $SL_{min}$3:Run spatial-temporal joint recovery inside coalitions with $SL_{min}$4:
**for** $\left(J=1;J\leq N_{Col};J++\right)$ **do**
5: **if** $SL_{Col_{j}}>\theta$ **then**6:  Use the common sparsity profile from coalition with $SL_{min}$ as input7: **end if**8: Run spatial-temporal joint recovery algorithm9: **end for**


## 8. Performance Evaluation

In order to evaluate our solution, we first analyse the impact of different tranform basis on compressive sensing. Then, we compare the distributed compression nature with centralized compressive sensing methods. We then compare our solution with other compressive sensing methods.

### 8.1. Assumption

For simulation, we have three different kinds of nodes: base station, coalition coordinator and sensor nodes. The base station groups these sensor nodes into different coalitions and assigns one coalition coordinator for each coalition. The coalition coordinator is responsible for transferring the coalition information to the base station. The base station is responsible for forming coalitions and running the optimal scheduling algorithm, which schedules communication in the network. Thus, there are no collisions in the network within or between coalitions. We transfer data by multi-hop communication, and each sensor node knows its geographical location and its connectivity radius. Two nodes are connected if and only if they are located in the communication range of each other. Coalition coordinator nodes are powerful nodes that act as aggregation centres.

We use a real dataset collected by Lausanne Urban Canopy Experiment (LUCE) wireless sensor network deployment at EPFL LUC [41]. This dataset gathers real temperature data using 64 ambient temperature sensor nodes. The sensor type is SHT75 sensor Sensirion. This dataset is sparse, which made it a very interesting dataset to evaluate recent compressive sensing solutions [42,43,44,45].

Our simulation is implemented in a MATLAB R2018a and we have used the toolbox provided for Bayesian compressive sensing in [38].

### 8.2. Impact Analysis of Transform Basis on Compressive Sensing Performance

**Figure 8:**
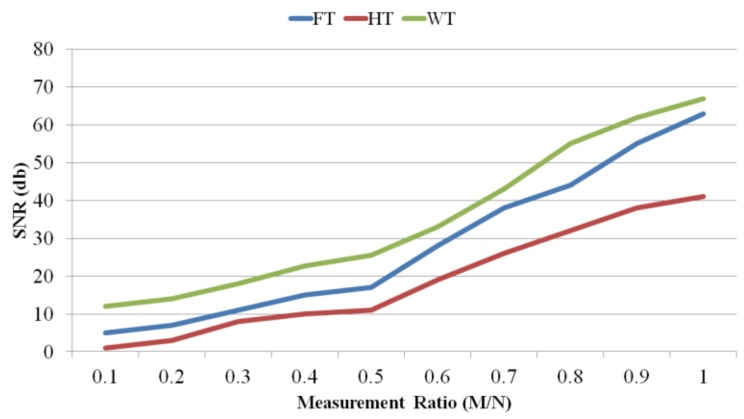
Comparing HT, WT, and FT in terms of SNR.

We analyse the effect of HT, WT (including Haar WT, Symlet WT, Coiflet WT, and Daubichi WT), and FT on the signal reconstruction accuracy. In order to compare the signal reconstruction accuracy, we use two metrics: SNR and normalized root mean square error (NRMSE). Figure 8 illustrates the comparison between HT, WT and FT in terms of SNR.

**Figure 9:**
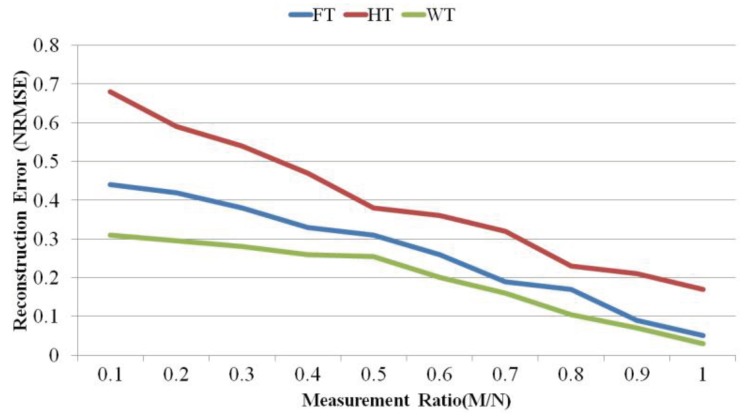
Comparing HT, WT, and FT in terms of signal reconstruction error.

As the figure shows, WT performs the best. Since WT is able to provide time and frequency information about the signal, it provides better sparsity than the other transform bases. Comparing them in terms of signal reconstruction error, as illustrated in Figure 9, shows that HT and WT have the worst and the best accuracy, respectively.

**Figure 10:**
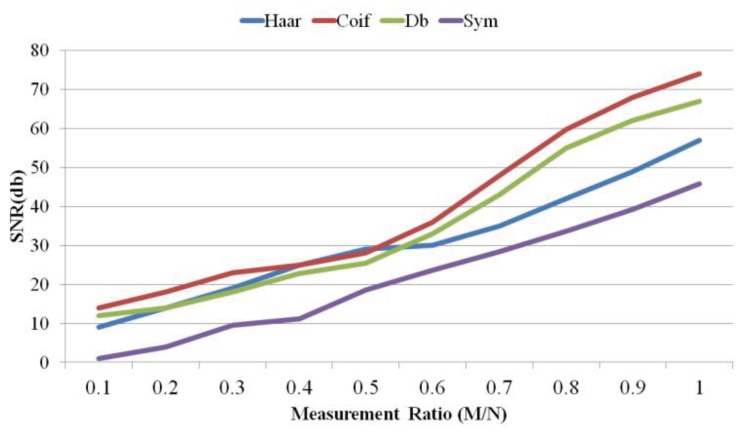
Comparing Haar WT, Symlet WT, Coiflet WT, and Daubichi WT in terms of SNR.

**Figure 11:**
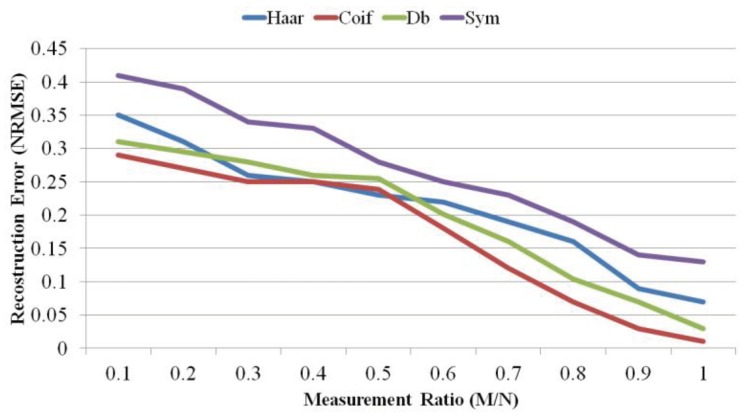
Comparing Haar, Symlet, Coiflet, and Daubichi transforms in terms of reconstruction error.

Because WT shows the best performance, we further analyze a number of transform sub-types. Figure 10 and Figure 11 illustrates the comparison between Haar WT, Symlet WT, Coiflet WT, and Daubichi WT. As we see, most of the time, Coiflet WT performs the best in terms of signal reconstruction error (measured as NRMSE) and SNR. Symlet WT performs the worst. However, when the measurement ratio is between 0.4 and 0.5, Haar WT shows better performance.

### 8.3. Comparing Distributed and Individual Compressive Sensing

The first part of the performance evaluation section investigates and compares distributed compressive sensing and individual compressive sensing approaches.

Typical signals measured by wireless sensor networks are sparse signals consisting of a common sparse component and different innovation components. Reconstructing these sorts of signals using joint sparse signal recovery models is more efficient than individual signal recovery models. It ultimately reduces the energy consumption and prolongs network lifetime.

**Figure 12:**
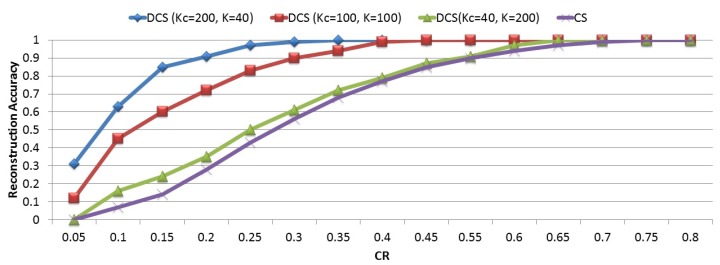
Reconstruction accuracy in terms of different common sparsity levels.

For this simulation, we change the number of common and innovative parts. As depicted in Figure 12, distributed compressive sensing provides accurate reconstruction with fewer measurements. On the other hand, increasing the number of common sparsity parameters provides better reconstruction accuracy. When the number of the common sparsity level is minimized, its performance approaches that of a separate compressive sensing method.

### 8.4. Comparing with Other Compressive Sensing Based Methods

In this section, we compare the proposed spatial-temporal compressive sensing approach with the existing data compression techniques. The approaches used in the comparison are Bayesian compressive sensing (BCS) [46], clustered spatio-temporal Bayesian compressive sensing (STBCS) [47], temporal belief-propagation based compressive sensing (TBCS) [6], orthogonal matching pursuit (OMP) based compressive sensing [48] and spatial Bayesian compressive sensing (SBCS) [49].

#### 8.4.1. Data Accuracy

**Figure 13:**
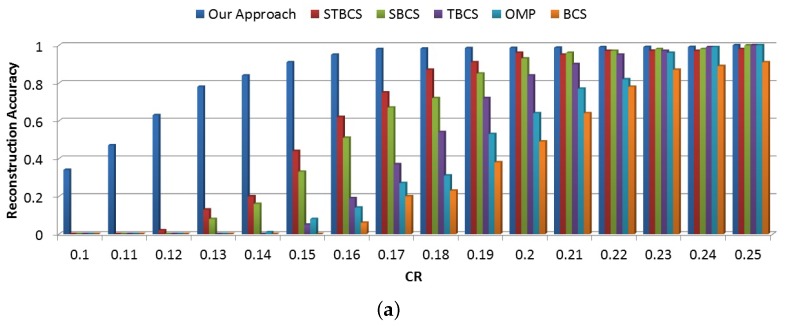

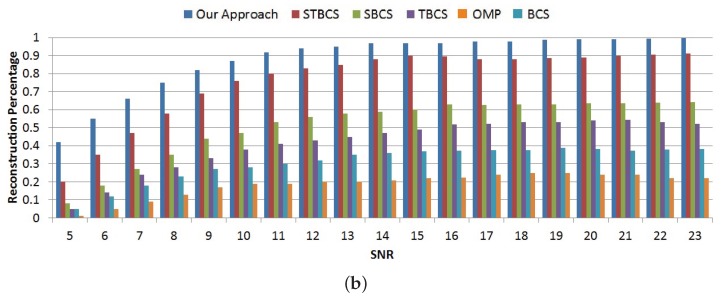
(**a**) Reconstruction accuracy percentage for different compression ratios. (**b**) Reconstruction accuracy percentage for different SNRs.

In order to measure the data accuracy, we first compare the accurate reconstruction percentage for the different data compression values. As Figure 13a indicates, our approach provides accurate data reconstruction with a minimum number of measurements. The main reason for this performance is the value of information gained using the coalition-formation method. Our approach tries to group sensor nodes based on their sparsity similarity, which leads to transmitting more information with a lower number of measurements. However, increasing the compression ratio decreases the gap between our approach and other methods. On the other hand, our approach adjusts the number of measurements based on the signal sparsity level, which removes redundant data transmission. As seen in Figure 13a, for more compression ratios, the reconstruction accuracy is almost the same.

An important parameter in the reconstruction procedure is the level of SNR. Figure 13b represents the reconstruction accuracy based on the SNR parameter. For lower SNR values, our approach represents better reconstruction accuracy. When increasing the SNR value, the STBCS model provides a similar level of accuracy but still lower than in our method. At SNR levels higher than 19, our method produces almost prefect reconstruction. Among other methods, the STBCS technique is the only one which shows comparable performance.

#### 8.4.2. Number of Transmission

**Figure 14:**
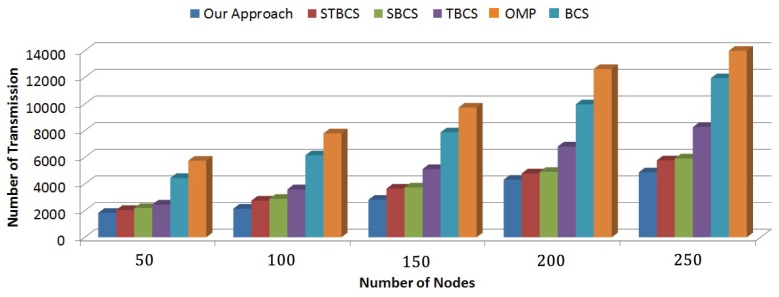
Number of data transmission versus number of sensor nodes.

One of the most important parameters affecting energy consumption is the number of data transmissions. The number of sensor nodes participating in data transmission is used to measure the number of transmissions. Figure 14 represents the number of transmissions for different compression techniques by changing number of sensor nodes.

For a network with a lower number of sensors, our approach has a minimum number of transmissions, but this number is comparable with STBCS and SBCS approaches. As the number of sensor nodes increases, the difference in the number of transmissions becomes more significant. This graph also demonstrates the scalability of our method. Our approach localizes sensor nodes based on the similarity of their sparsity levels in separate coalitions. For each coalition, it then curtails the number of transmissions by lessening the number of active sensor nodes participating in data transmission and adjusting the number of measurements to the sparsity level. Therefore, our approach involves a minimum number of transmissions.

#### 8.4.3. Energy Consumption

**Figure 15:**
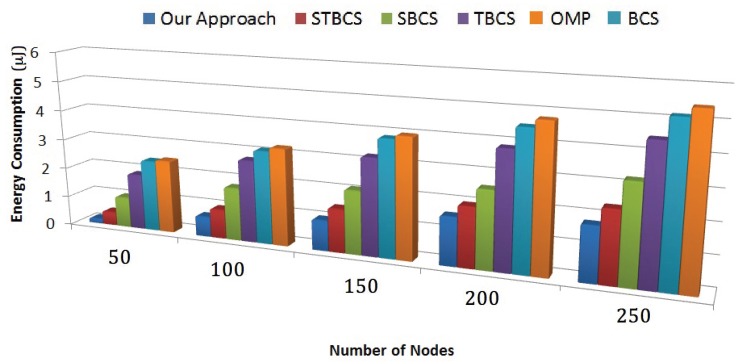
Energy consumption.

In this section, we compare energy consumption parameters among the different methods. As we can see in Figure 15, our approach provides the best energy resource consumption performance among the approaches compared. Our approach adapts both the number of measurements and the number of active sensor nodes to minimize the energy expenditure. The STBCS and SBCS are the second and third best approaches, which provide performance somewhat more comparable to our method than the other methods do. The main common feature of these three approaches is grouping sensor nodes and localizing sensor node measurements.

The means of grouping sensor nodes and gathering measurements from each group are the main difference among these methods. Since our approach groups the sensor nodes with reference to sparsity similarity, the number of active nodes is diminished. Moreover, it adjusts the number of measurements to the sparsity level, which removes the redundancy in data gathering. STBCS uses a clustering approach to group sensor nodes, but its grouping mechanism is not as accurate as that of our method. In addition, it does not consider adaptability to the number of measurements. Moreover, TBCS, BCS and OMP do not include localization of data gathering, which leads to greater exhaustion of energy.

#### 8.4.4. Trade-Off between Energy and Accuracy

We study the trade-off between network energy usage and data accuracy. To do so, we investigate energy consumption in terms of normalized network lifetime, while for accuracy we consider the error between the reconstructed data and real data.

**Figure 16:**
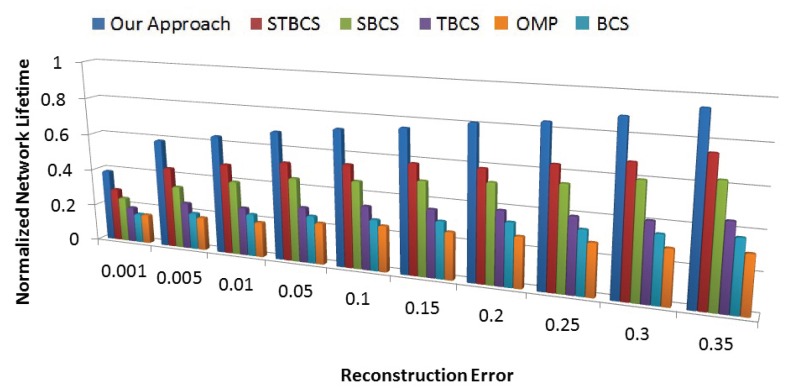
Normalized network lifetime versus reconstruction error trade-off.

As we can see in Figure 16, our approach provides approximately 40% network lifetime at a minimum error level, while with less accurate data it provides higher percentages of lifetime. Adapting both the number of measurements and the number of active sensor nodes to the signal sparsity level is the main contribution of our approach to prolong network lifetime. In addition, spatial-temporal correlation-based coalition formation provides sufficient similarity among sensor node measurements, which leads to less error in the recovered data.

## 9. Conclusions

We have presented a distributed compressive sensing scheme that takes the advantage of spatial temporal correlation of sensor node readings to achieve an energy efficient data collection. Spatial correlation among sensor nodes and sparsity distribution of signals over the network is used to group sensor nodes in the coalitions. The proposed coalition-formation method is represented by a block diagonal measurement matrix in which each diagonal entity corresponds to one of the coalitions. The localized spatial temporal correlation inside each coalition builds an efficient measurement matrix that efficiently schedules sensor nodes and balances the data compression and transmission load over the coalition. Upon receiving compressed data in the base station, a two-step belief propagation based signal recovery is implemented to reconstruct the original signal. Having a two-step signal recovery allows the base station to find the common coefficient of original signal among coalitions and inside coalition. Therefore, it has enough data to recover data per sensor node level, which increases data reconstruction accuracy. Coalition-based data gathering is used to scale down transmission costs, while the number of measurements is minimized by scheduling sensor nodes and adjusting their measurement rate. Our simulation on a real world data set proves our findings and shows more reduction on the number of data measurements and compressibility as well as the ability to improve data reconstruction quality.

This paper has also presented a number of transform bases and analyzed their effect on compressive sensing performance in terms of SNR and signal reconstruction error. Since most signals acquired by wireless sensor networks are non-stationary, we considered data collected from a bridge monitoring application as a case study for this impact analysis. The comparison of simulation results using FT, HT and WT demonstrates that WT has the best performance when applying compressive sensing to this case study. Coiflet WT, Daubichi WT, Symlet WT, and Haar WT, the sub-families of WT, were also studied and analysed in terms of SNR and NRMSE.

## Figures and Tables

**Table 1:** Notations.

N	number of sensor nodes	$N_{C}$	number of coalitions
X	signal	Y	measurement
J	coalition index	C	coalition matrix
Φ	basis function	$E_{Co}$	energy cost
Dist(i,j)	distance between nodes *i*,*j*	CR	correlation metric
SCD	sparsity cover degree	$Col_{j}$	coalition ID
$n_{i}$	node index	$l_{i,j}$	link ID
CC	coalition coordinator	$N_{ANode}$	number of active nodes
SN	set of sensor nodes	ST	set of sampling times
M	number of measurements	K	sparisity degree
Comm	number of common non-zero elements	SL	similarity level
CSP	common sparsity profile	CR	compression ratio
SNR	signal to noise ratio	$P_{j}$	number of active nodes in coalition *j*
